# Randomized Controlled Trial of Salt and Amiloride on Vascular Stiffness in ADPKD

**DOI:** 10.1016/j.ekir.2026.107110

**Published:** 2026-09-23

**Authors:** Anahita Noruzi, Laixi Xue, Wesley J. Visser, Robert Zietse, Joep van der Leeuw, Jeroen van der Net, Darius Soonawala, Sanne C.M. Boeters, A.H. Jan Danser, Ewout J. Hoorn, Mahdi Salih

**Affiliations:** 1 Division of Nephrology and Transplantation, Department of Internal Medicine, Erasmus Medical Center, Rotterdam, The Netherlands; 2 Division of Dietetics, Department of Internal Medicine, Erasmus Medical Center, Rotterdam, The Netherlands; 3 Department of Internal Medicine, Franciscus Hospital, Rotterdam, The Netherlands; 4 Department of Internal Medicine, Albert Schweitzer Hospital, Dordrecht, The Netherlands; 5 Department of Internal Medicine, Haga Hospital, The Hague, The Netherlands; 6 Division of Pharmacology and Vascular Medicine, Department of Internal Medicine, Erasmus Medical Center, Rotterdam, The Netherlands

**Keywords:** amiloride, hypertension, salt, vascular stiffness

## Abstract

**Introduction:**

Patients with autosomal dominant polycystic kidney disease (ADPKD) often develop early vascular stiffening and hypertension. Dietary sodium may worsen vascular stiffness via epithelial sodium channel (ENaC) activation, and ENaC inhibition might mitigate this, but this remains untested in ADPKD. We hypothesized that dietary sodium loading increases vascular stiffness in ADPKD and that the ENaC inhibitor amiloride attenuates this effect.

**Methods:**

In this double-blind, randomized, placebo-controlled trial, adults with ADPKD and estimated glomerular filtration rate (eGFR) ≥60 ml/min per 1.73 m^2^ completed a 2-week low-salt run-in (≤ 3.5 g/d), then were randomized 1:1 to oral sodium chloride capsules (6 g/d) or placebo for 2 weeks, followed by a 2-week open-label amiloride (20 mg/d) add-on phase in both arms. The primary outcome was pulse wave velocity (PWV). Secondary outcomes included 24-hour blood pressure (BP) and biochemical parameters.

**Results:**

Thirty-seven participants (mean age 39 ± 11 years, eGFR 89 ± 19 ml/min per 1.73 m^2^) were randomized to salt (*n* = 19) or placebo (*n* = 18). Salt supplementation increased PWV (6.5 ± 0.9 m/s to 6.8 ± 1.1 m/s, *P* = 0.02) and 24-hour systolic BP (133 ± 13 mm Hg to 138 ± 15 mm Hg, *P* = 0.02). Amiloride did not reduce PWV in either group, but decreased systolic BP only in the low-salt group (135 ± 11 to 130 ± 13 mm Hg, *P* = 0.03). Plasma potassium increased modestly in both groups following amiloride, without hyperkalemia.

**Conclusion:**

In this small, short-term trial, dietary sodium significantly increased vascular stiffness and BP in early ADPKD, and amiloride significantly lowered BP only under low-salt conditions. These findings support dietary sodium restriction as a practical strategy for BP and vascular protection in early ADPKD.

## Graphical abstract

**Figure:**
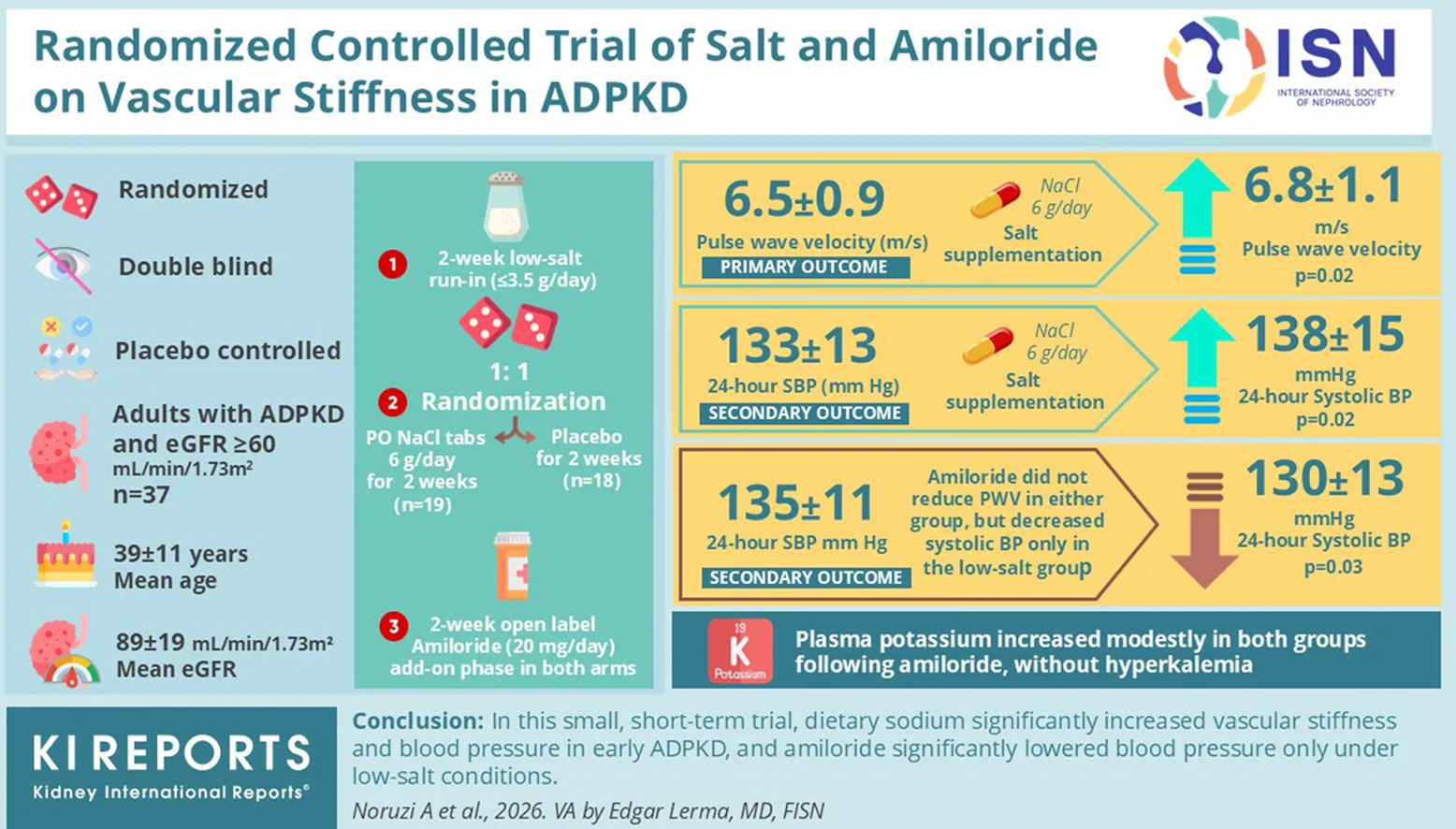

ADPKD is the most common inherited kidney disease and is predominantly caused by pathogenic variants in the *PKD1* and *PKD2* genes.^1^ These genes encode polycystin-1 (PC-1) and polycystin-2 (PC-2), which form a mechanosensitive complex involved in tubular and vascular cell signaling. Dysfunction of this complex leads to the progressive development and expansion of kidney cysts, resulting in kidney function decline and in most cases kidney failure.^2^

Hypertension is a hallmark of ADPKD and typically develops early in the disease course, often before the onset of kidney function decline.^3^ Early-onset hypertension is associated with a more rapid loss of kidney function and increased cardiovascular mortality.^4^ Patients with ADPKD show impaired endothelium-dependent vasodilation and reduced nitric oxide release even when normotensive and kidney function is preserved, reflecting altered mechanosensitivity of the PC-1/PC-2 complex in vascular endothelial cells to shear stress.^5^ Endothelium-specific deletion of *PKD1* in mice reproduces this phenotype, causing impaired flow-mediated dilation and hypertension,^6^ establishing a plausible ADPKD-specific vascular substrate for hypertension independent of kidney function decline.

Separately, in the general population, high dietary sodium intake impairs endothelial function and increases vascular stiffness through activation of ENaC in vascular endothelial cells,^7^, ^8^, ^9^ promoting endothelial cell rigidity and reduced nitric oxide bioavailability.^10^ Whether this salt-ENaC pathway interacts with the polycystin-dependent vascular abnormalities described above is unknown—no study has directly examined ENaC expression or activity in the ADPKD vasculature. Given that ADPKD vessels are already sensitized to endothelial dysfunction through a separate mechanism, dietary sodium loading could plausibly unmask or exacerbate vascular stiffness in this population even without ADPKD-specific ENaC dysregulation, but this has not been tested.

Despite the known early vascular vulnerability in ADPKD, interventional human data on the effects of dietary sodium intake and ENaC inhibition on vascular stiffness in ADPKD are lacking. In particular, it is unknown whether salt intake worsens vascular stiffness in patients with early ADPKD on top of their polycystin-related endothelial vulnerability, and whether pharmacologic ENaC inhibition with amiloride can counteract such salt-induced vascular changes. Determining the effects of these interventions is needed to refine additional strategies for BP control and cardiovascular risk reduction in this population.

Building on this rationale, we hypothesized that dietary sodium intake increases vascular stiffness in patients with ADPKD and that pharmacological inhibition of ENaC with amiloride attenuates these effects. To test this hypothesis, we conducted the treatment of vascular stiffness in autosomal dominant polycystic kidney disease (TRAMPOLINE) study, an investigator-initiated, randomized, double-blind, placebo-controlled clinical trial with an open-label amiloride phase. The primary objective was to evaluate the effect of controlled high-salt versus low-salt intake on PWV, a validated measure of arterial stiffness, in patients with ADPKD and preserved kidney function. Secondary objectives were to assess the impact of amiloride on PWV and ambulatory BP during low or high salt intake.

## Methods

### Study Design

The TRAMPOLINE study is a single-center, double-blind, randomized, placebo-controlled trial with a subsequent open-label amiloride phase, performed at the Erasmus University Medical Center, Rotterdam, The Netherlands. Patients were recruited from March 2022 until May 2025. The total duration of the study was seven weeks and consisted of 5 in-person visits (Figure 1). All patients followed a low-salt diet (≤ 3.5 g/d) for 6 weeks. To increase adherence to dietary sodium restriction, patients received dietary counseling from a renal dietitian at the start of the low-salt diet, after 1, 3, and 5 weeks. In addition to dietary counselling, patients received salt-free bread for the whole intervention period. All participants first completed a standardized 2-week low-salt run-in phase with washout of all antihypertensive medications, which remained withheld for the duration of the study. Participants received standard care throughout the study. After this period, eligible participants were randomized in a 1:1 ratio to receive either oral sodium chloride capsules (6 g/d) or matching placebo for 2 weeks, using permuted block randomization stratified by age and body mass index (BMI). Allocation was performed via a secure system, managed by the pharmacy. Participants, treating clinicians, investigators, and outcome assessors remained blinded to treatment allocation during the placebo or salt phase. Capsules were indistinguishable in appearance, weight, and packaging. To preserve blinding, 24-hour urinary sodium and chloride results were concealed from investigators, study staff, and participants until completion of the study. This double-blind phase was followed by an additional 2-week open-label phase in which all participants received amiloride 20 mg once daily while continuing their assigned capsules. Study medication was dispensed by the hospital pharmacy according to the randomization schedule. Participants were instructed to take the assigned capsules daily for 2 weeks, and adherence was assessed by capsule counts and supported by 24-hour urinary sodium excretion after de-blinding. The trial was designed to evaluate the effects of salt supplementation and ENaC inhibition on arterial stiffness as assessed by PWV. The study was conducted according to the Declaration of Helsinki and International Conference on Harmonization–Good Clinical Practice guidelines. The institutional medical ethics committee of Erasmus University Medical Center approved the protocol (MEC-2021-0593). All participants provided written informed consent. The study is registered at ClinicalTrials.gov (NCT05228574).

**Figure 1:**
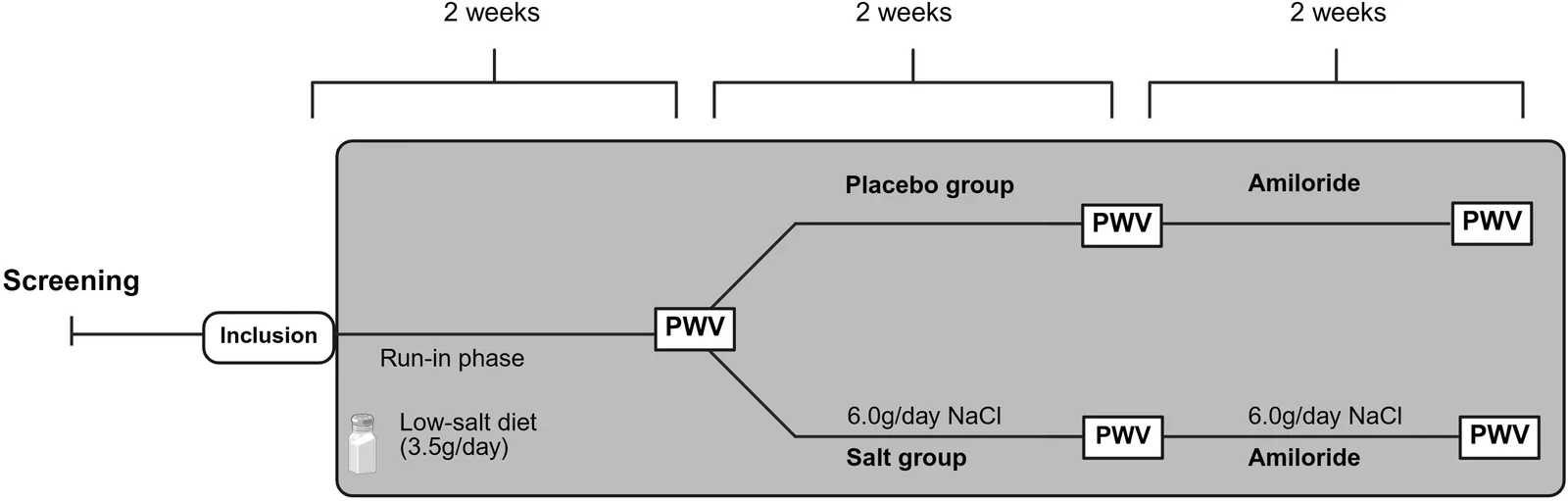
Schematic overview of the study design. After inclusion, participants underwent a 2-week washout period where all antihypertensive drugs were discontinued and they initiated the low-salt diet (grey area). Participants were then randomized to receive either placebo or salt capsules, and finally, open-label amiloride. Pulse wave velocity (PWV) measurements were performed after the washout period (baseline), after initiation of capsules (placebo/salt), and after amiloride use. Blood and urine analyses were performed concurrently with each PWV assessment.

### Participants

Adults aged ≥18 years with a diagnosis of ADPKD according to the modified Ravine criteria^11^ and an eGFR of ≥60 ml/min per 1.73 m^2^ were eligible. Key exclusion criteria were as follows: uncontrolled hypertension, defined as an office BP of ≥160/ ≥90 mm Hg with or without antihypertensive treatment, use of ≥3 antihypertensive medications, antihypertensive treatment for any other treatment indication than hypertension (e.g., cardiac arrhythmia), plasma potassium levels > 5.5 mmol/l measured within last 6 months, history of liver disease (excluding liver cysts because of ADPKD), history of heart failure (cardiac ejection fraction < 35%), diabetes mellitus, active infection or antibiotic therapy, immunosuppressive therapy within the last year, concomitant use of drugs that could influence BP and/or disease progression (tolvaptan, nonsteroidal anti-inflammatory drugs, and chemotherapy), and pregnancy or unwillingness to adhere to reproductive precautions during the study.

### Primary Outcome

The primary outcome was arterial stiffness, quantified by PWV measured at the following 3 predefined time points: (i) after the 2-week low-salt run-in (baseline), (ii) after 2 weeks of placebo or salt capsules, and (iii) after 2 weeks of amiloride add-on therapy. PWV was measured using the Mobil-O-Graph device (IEM, Stolberg, Germany), which records 24-hour brachial pulse waves and derives aortic PWV using validated transfer functions.

### Secondary Outcomes

Secondary outcomes included 24-hour ambulatory systolic and diastolic BP, plasma electrolytes, urea, creatinine, and bicarbonate, and 24-hour urine sodium, potassium, chloride, urea, osmolality, pH, and albumin. 24-hour urinary sodium and chloride excretion served as biochemical indicators of adherence to the assigned sodium interventions. Furthermore, circulating markers related to the renin–angiotensin–aldosterone system and endothelial function were assessed, including plasma renin (IRMA; Beckman Coulter Immunotech, Prague, Czech Republic; sensitivity 0.81 pg/mL), aldosterone (radioimmunoassay; Beckman Coulter Immunotech, Prague, Czech Republic; sensitivity 12 pg/mL), intercellular adhesion molecule-1 (ICAM-1; Human ICAM-1/CD54 Allele-specific Immunoassay, Catalog Number DCD540, Bio-Techne/R&D Systems), vascular cell adhesion molecule-1 (VCAM-1; Human VCAM-1/CD106 Immunoassay, Catalog Number DVC00, Bio-Techne/R&D Systems), endothelin-1 (ET-1; Endothelin-1 Immunoassay, Catalog Number DET100, Bio-Techne/R&D Systems), and vascular endothelial growth factor (VEGF; Human VEGF Immunoassay, Catalog Number DVE00, Bio-Techne/R&D Systems). Ambulatory BP measurements were performed using the Mobil-O-Graph according to National Institute for Health and Care Excellence recommendations,^12^ with measurements every 30 minutes during daytime (08:00–22:00) and every 60 minutes during nighttime (22:00–08:00). A minimum of 14 daytime readings was required for data validity.

### Adverse Events

Adverse events were defined as any undesirable experience occurring during the study, regardless of whether the event is related to the study intervention. Serious adverse events included events resulting in death, life-threatening conditions, or hospitalization, with the exception of elective hospitalizations. Suspected unexpected serious adverse reactions were defined as serious, unexpected adverse reactions with a reasonable possibility of being caused by the intervention, based on the nature and severity of the event.

### Sample Size Calculation

The sample size was based on detecting differences in PWV across 3 prespecified within-subject comparisons. The smallest expected mean change was a 1.15 m/s reduction in PWV with amiloride, assuming an SD of 1.7 m/s (derived from data in young individuals with preserved kidney function.^13^^,^^14^ To ensure adequate power under conservative assumptions, a 2-sided α of 0.017 was used for the sample size calculation. A total of 52 participants was required for 80% power using a 2-sided paired t-test. To account for potential dropouts, 54 patients were planned. Because the observed variability in PWV was lower than anticipated in the first part of the study (SD = 0.44 m/s), a blinded reassessment of the sample size was performed by an independent statistician. This reassessment was not specified in the original protocol or statistical analysis plan but was triggered by the observation that pooled PWV variability across all participants, visible because outcome measurements, unlike treatment allocation were not blinded, was substantially lower than the value used in the original power calculation. This reassessment was based solely on the updated estimate of variability, without access to treatment allocation or effect size information. Based on the revised calculation, a total of 16 participants was required, and the predefined sample size requirement was met; recruitment was therefore stopped early after inclusion of 40 participants. Ethical approval for the amended sample size was obtained.

### Statistical Analysis

The primary analysis population consisted of all randomized participants who had at least 1 postrandomization PWV measurement (modified intention-to-treat approach). The safety population included all individuals who received at least 1 dose of either sodium chloride or placebo capsules or at least 1 dose of amiloride. Continuous variables were visually inspected for normality using histograms and Q-Q plots, supplemented by the Shapiro-Wilk test. Depending on their distribution, data were summarized as mean ± SD or median with Q1-Q3 range. Categorical variables were summarized as counts and percentages. Between-group comparisons of continuous variables (salt vs. low-salt) were made using independent-samples *t* tests for normally distributed data and the Mann–Whitney U test for non-normally distributed data. Within-group changes over time were evaluated using paired *t* tests for normally distributed variables and the Wilcoxon signed-rank test for non-normally distributed variables. Categorical variables were compared using chi-square tests, and Fisher exact test was applied when expected cell counts were below 5. A 2-sided *P* < 0.05 was considered statistically significant. Missing data were handled by complete-case analysis, consistent with the prespecified analytic plan and the limited sample size. All statistical analyses were performed using R version 4.3.3 (2024-02-29; R Project, Vienna, Austria).

## Results

### Participant Flow and Baseline Characteristics

A total of 484 patients were screened for eligibility, of whom 40 were enrolled and 37 were subsequently randomized to the salt group (*n* = 19) or the low-salt (placebo) group (*n* = 18; Figure 2). Fifteen participants in the salt group and 17 in the low-salt group completed all study visits. Baseline characteristics after completion of the run-in phase are presented in Table 1. Mayo Imaging Classification was available from routine clinical care for only 5 participants (atypical, 1E, 1C, 1B, and 1B). The 2 groups were comparable with respect to age, BMI, sex distribution, BP, PWV, kidney function, historical eGFR decline, and use of antihypertensive medications. A higher proportion of participants in the low-salt group had a history of smoking (56% vs. 11%), although cumulative exposure expressed as pack-years did not differ. Baseline 24-hour urinary sodium and chloride excretion indicated good adherence to the low-sodium diet in both groups. However, sodium and chloride excretion were significantly lower in the salt group (both *P* < 0.01; Figure 3). Additionally, plasma aldosterone was slightly lower at baseline in the low-salt group (median 188 vs. 280, whereas no difference between groups was observed in plasma renin levels (median 21.8 vs. 20.2).

**Figure 2:**
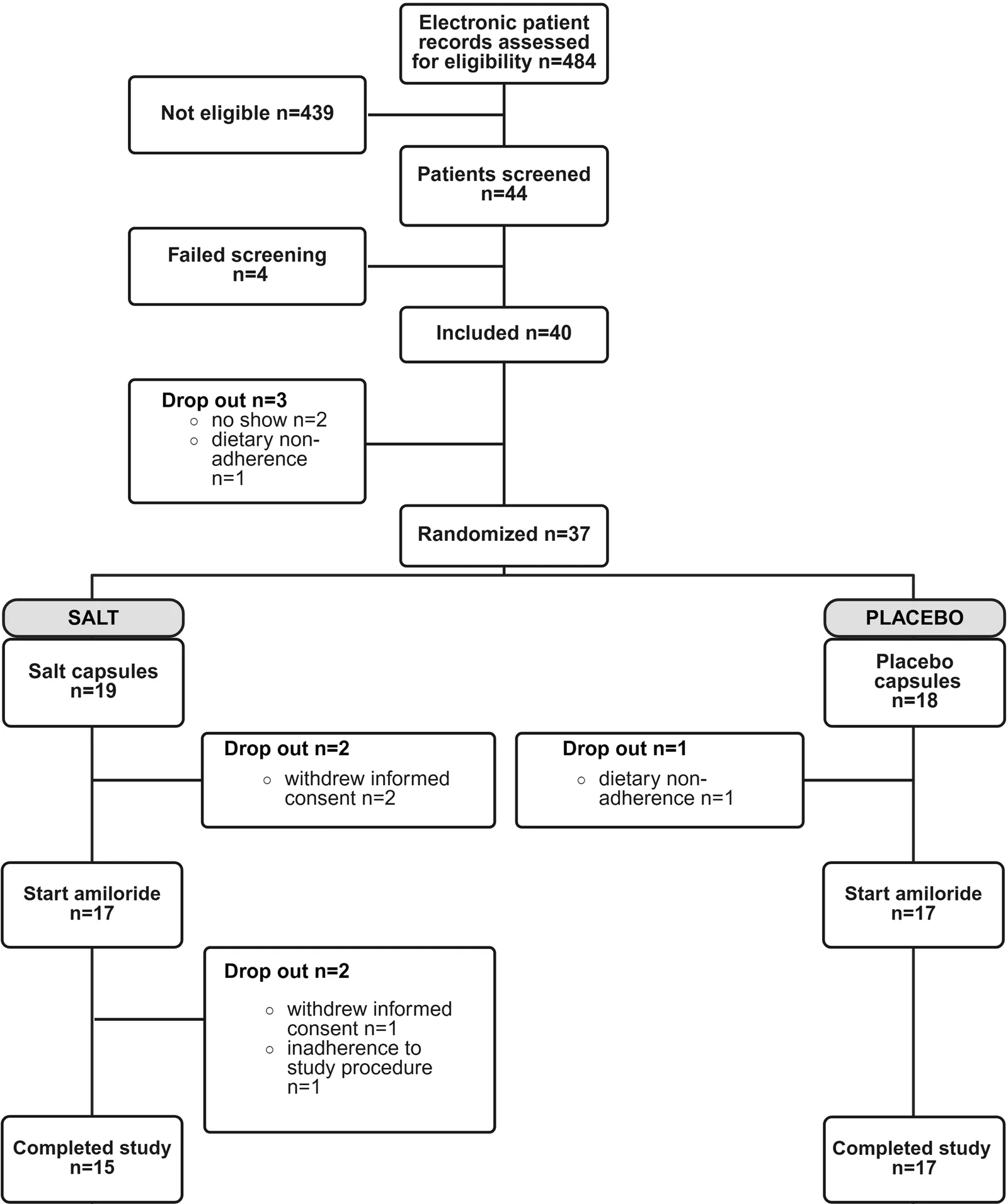
Study flowchart.

**Table 1:** Patient characteristics after the low-salt run-in phase (pre-randomization), stratified by intervention group (*N* = 37)

Patient characteristics	Total *N* = 37	Low salt group *n* = 18	Salt group *n* = 19
Age, yrs	39 ± 11	39 ± 12	39 ± 9
BMI, kg/m^2^	25.1 ± 4.2	25.4 ± 5.1	24.8 ± 3.4
Male sex, *n* (%)	8 (22%)	5 (28%)	3 (16%)
Pathogenic variant, *n*			
• No genetic analysis performed	22	11	11
• *PKD1* truncating	4	2	2
• *PKD1* nontruncating	5	5	0
• *PKD2*	6	0	6
• Other^a^	2	0	2
Systolic 24-h BP, mm Hg	134 ± 13	135 ± 13	133 ± 13
Diastolic 24-h BP, mm Hg	85 ± 8	84 ± 8	85 ± 9
Pulse wave velocity, m/s	6.6 ± 1.0	6.6 ± 1.1	6.5 ± 0.9
Antihypertensives used, *n*	19	12	7
• ACEi, *n*	7	3	4
• Angiotensin receptor blocker (ARB), *n*	5	4	1
• Calcium antagonists, *n*	4	2	2
• Other antihypertensive drugs, *n*	1	1	0
• Thiazide diuretics, *n*	2	2	0
History of smoking, *n* (%)	12 (32%)	10 (56%)	2 (11%)
Pack years among smokers^b^	7.5 (4.8–11.0)	9.0 (4.2–13.0)	6.0 (5.5–6.5)
Historic eGFR decline, ml/min per 1.73 m^2^/yr	−2.43 ± 2.36	−2.45 ± 1.99	−2.39 ± 2.88
Plasma measurements			
Creatinine, μmol/l	80 ± 17	79 ± 16	81 ± 18
eGFR, ml/min per 1.73 m^2^	89 ± 19	91 ± 20	86 ± 18
Urea, mmol/l	5.0 ± 1.9	5.0 ± 2.3	4.9 ± 1.4
Sodium, mmol/l	140 ± 3	140 ± 3	140 ± 2
Potassium, mmol/l	4.0 ± 0.3	4.1 ± 0.3	4.0 ± 0.3
Bicarbonate, mmol/l	26.8 ± 2.2	27.0 ± 2.4	26.7 ± 2.1
Chloride, mmol/l	103 ± 2	102 ± 3	103 ± 2
Renin, pg/ml^b^	21.6 (15.0-29.8)	21.8 (16.9–30.3)	20.2 (13.2–29.0)
Aldosterone, pg/ml^b^	224 (153-349)	188 (119–248)	280 (221–364)
24-h urine measurements			
Volume, ml	2326 ± 993	2446 ± 1127	2206 ± 855
pH	6.4 ± 0.6	6.5 ± 0.5	6.2 ± 0.6
Osmolarity, mOsm/kg	297 ± 155	300 ± 153	294 ± 162
Creatinine, mmol/d	12 ± 4	12 ± 4	12 ± 4
Urea, mmol/d	326 ± 143	353 ± 179	299 ± 93
Sodium, mmol/d	47 ± 22	57 ± 21	37 ± 20
Potassium, mmol/d	74 ± 31	80 ± 35	67 ± 26
Chloride, mmol/d	53 ± 23	64 ± 23	42 ± 17
Albumin, mg/d^b^	10 (10–32)	10 (10–35)	10 (10–28)

ACE, angiotensin converting enzyme; BMI, body mass index; eGFR, estimated glomerular filtration rate.

a *PKHD1* and *PKD1* truncating; *PKD2* and *IFT140*.

b Median (Q1–Q3).

**Figure 3:**
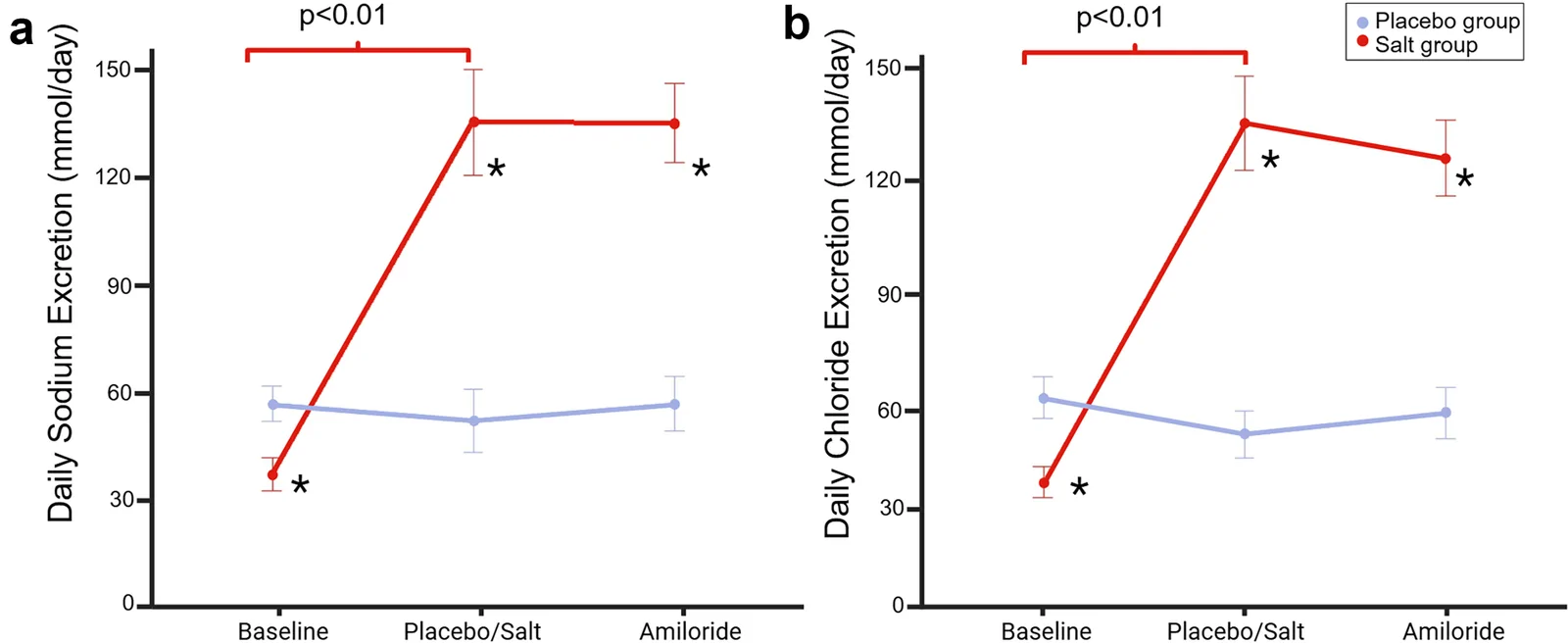
Changes in daily sodium (a) and chloride (b) excretion, stratified by group. Asterisks indicate between-group differences at each phase: ∗*P* < 0.01. Within group differences are shown in the graphs by brackets, corresponding to the group colors.

### Effects of Salt Intake

Twenty-four–hour urinary sodium and chloride excretion increased significantly in the salt group and remained unchanged in the low-salt group, confirming effective separation of salt intake during the randomized phase (Table 2, Figures 4 and 5). After 2 weeks of salt supplementation, PWV increased significantly in the salt group from 6.5 ± 0.9 m/s to 6.8 ± 1.1 m/s (*P* = 0.02), whereas no significant change occurred in the low-salt group (6.6 ± 1.1 m/s to 6.6 ± 1.0 m/s; *P* = 0.67). Twenty-four-hour systolic BP increased from 133 ± 13 mm Hg to 138 ± 15 mm Hg in the salt group (*P* = 0.02), with no significant change in the low-salt group. Diastolic BP remained unchanged in both arms.

**Table 2:** Clinical and biochemical values after initiation of salt or placebo capsules

Outcomes	Low salt group *n* = 17	Salt group *n*= 17	*P*-value
BP and PWV values			
24-h systolic BP, mm Hg	135 ± 11	138 ± 15	0.48
24-h diastolic BP, mm Hg	85 ± 9	87 ± 9	0.48
Pulse wave velocity, m/s	6.6 ± 1.0	6.8 ± 1.1	0.68
Plasma measurements			
Creatinine, μmol/l	80 ± 15	81 ± 21	0.69
eGFR, ml/min per 1.73 m^2^	89 ± 19	87 ± 22	0.75
Urea, mmol/l	5.1 ± 1.8	4.9 ± 1.3	0.71
Sodium, mmol/l	140 ± 3	141 ± 1	0.06
Potassium, mmol/l	4.1 ± 0.3	4.0 ± 0.2	0.34
Bicarbonate, mmol/l	27.6 ± 1.8	25.9 ± 2.1	0.02
Chloride, mmol/l	102 ± 3	105 ± 2	< 0.01
Renin, pg/ml^a^	16.77 (13.75–26.32)	11.29 (7.59–13.58)	0.02
Aldosterone, pg/ml^a^	229 (139–312)	169.47 (125–198)	0.05
24-h urine measurements			
Volume, ml	2519 ± 662	2428 ± 999	0.76
pH	6.3 ± 0.5	6.2 ± 0.4	0.82
Osmolarity, mOsm/kg	219 ± 42	363 ± 186	0.01
Creatinine, mmol/d	11 ± 4	13 ± 5	0.14
Urea, mmol/d	298 ± 125	342 ± 121	0.30
Sodium, mmol/d	52 ± 32	135 ± 55	< 0.01
Potassium, mmol/d	65 ± 27	76 ± 23	0.26
Chloride, mmol/d	54 ± 22	135 ± 46	< 0.01
Albumin, mg/d^a^	15 (10–30)	20 (10–38)	0.72

eGFR, estimated glomerular filtration rate; PWV, pulse wave velocity.

a Median (Q1–Q3).

**Figure 4:**
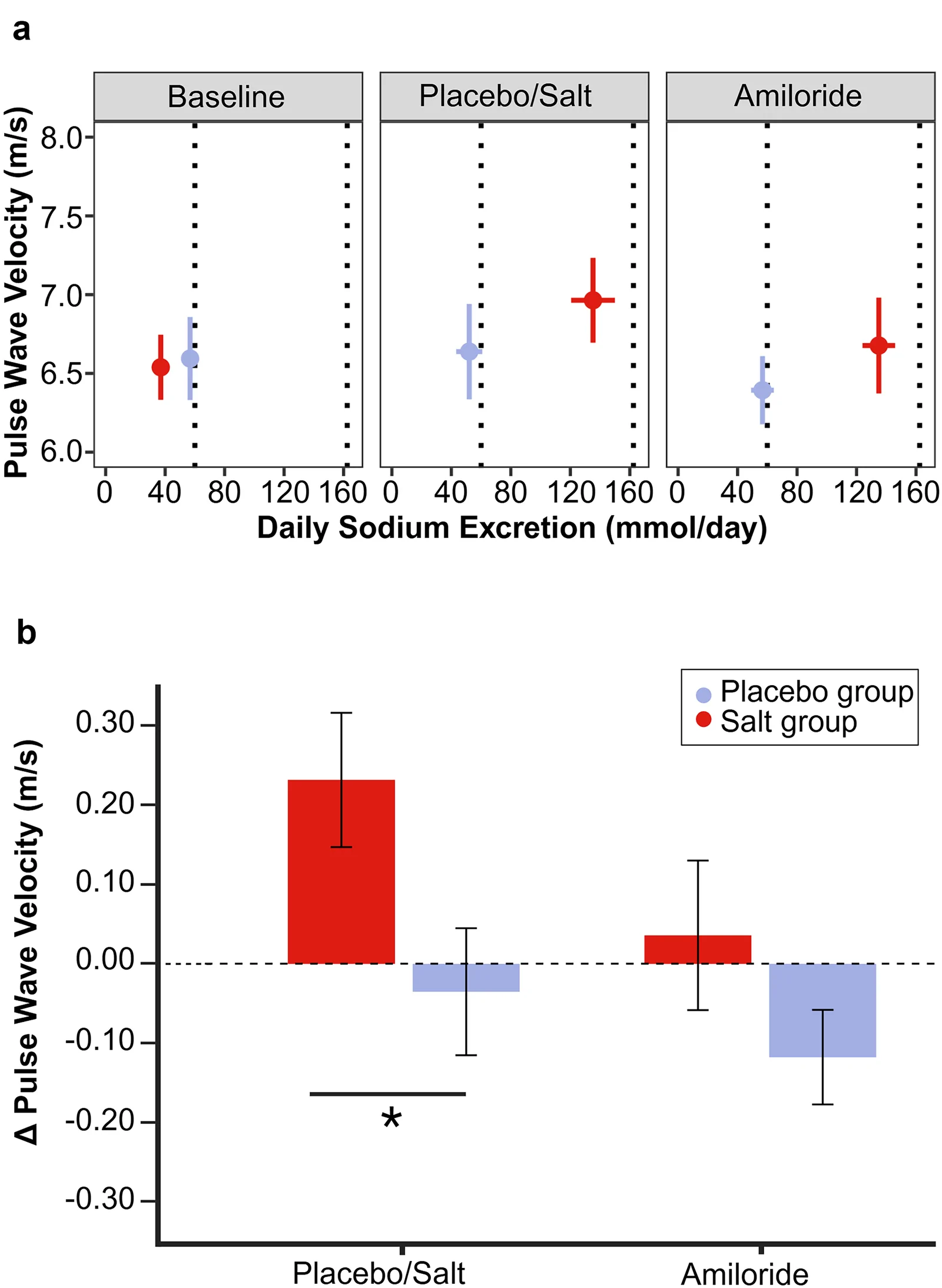
Changes in pulse wave velocity. (a) Graph showing the mean ± SEM of pulse wave velocity and daily sodium excretion at baseline, after intervention and amiloride treatment, during low- and high-salt diet (reflected by 24-hour urinary sodium excretion, X-axis). Dotted lines represent aimed salt intake (3.5 and 9.5 g/d). (b) Graph showing the mean ± SEM change in baseline pulse wave velocity, stratified by group. Asterisks indicate between-group differences ∗*P* = 0.03.

**Figure 5:**
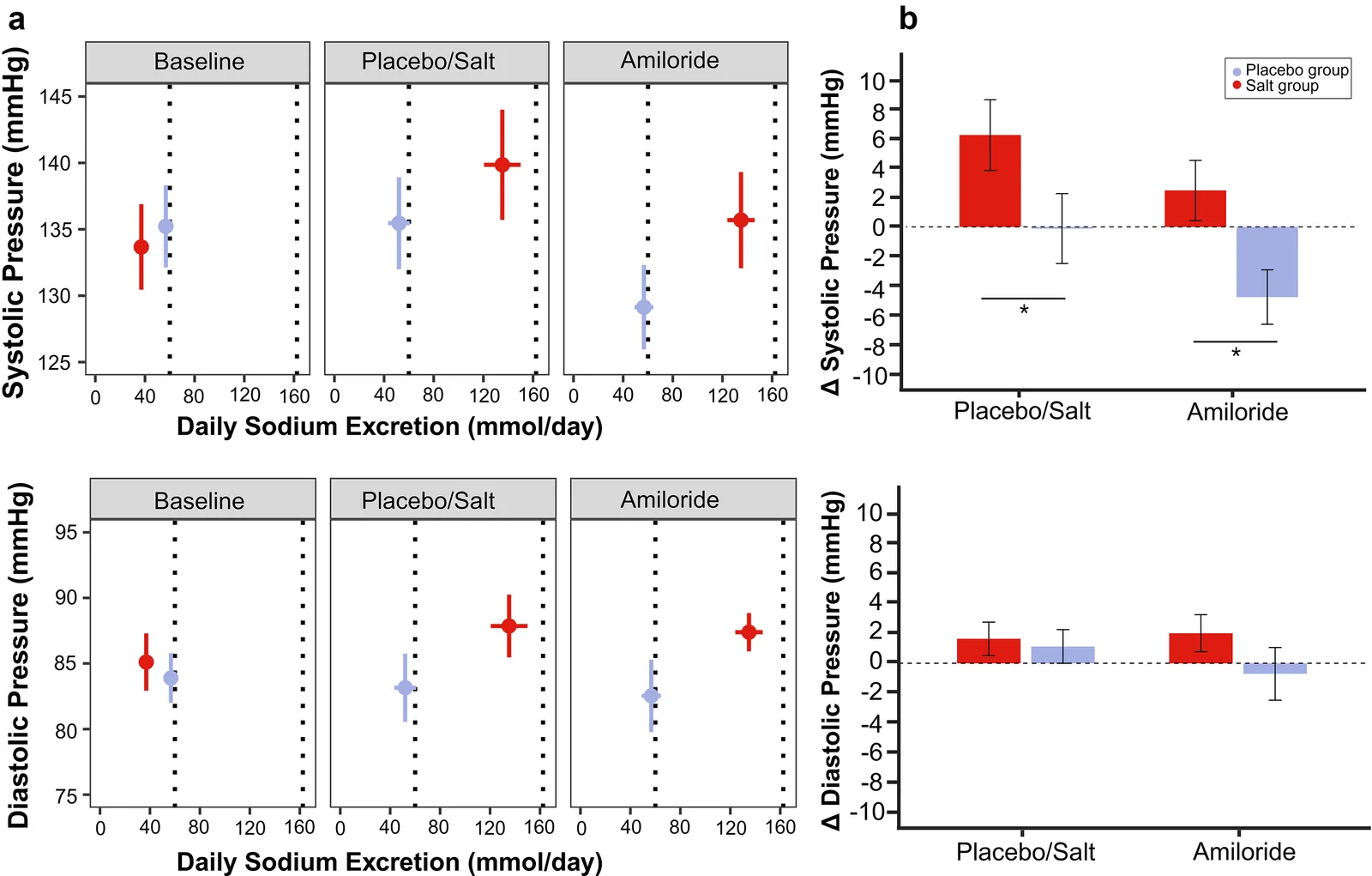
Changes in 24-hour BP. (a) Graph showing the mean ± SEM of systolic and diastolic BP and daily sodium excretion at baseline, after intervention and amiloride treatment, during low- and high-salt diet (reflected by 24-hour urinary sodium excretion, X-axis). Dotted lines represent aimed salt intake (3.5 and 9.5 g/d). (b) Graph showing the mean ± SEM change in baseline systolic and diastolic BP, stratified by group. Asterisks indicate between-group differences ∗*P* < 0.05.

### Effects of Amiloride

Tables 3 and 4 summarize all within-group changes during the amiloride phase; between group differences are shown in Supplementary Tables S1 and S2. After the initiation of amiloride, PWV and 24-hour systolic and diastolic BP remained unchanged in the salt group (6.8 ± 1.1 m/s to 6.6 ± 1.1 m/s; *P* = 0.10, 138 ± 15 mm Hg to 135 ± 13 mm Hg; *P* = 0.30, and 87 ± 9 to 87 ± 5 mm Hg; *P* = 0.32, respectively). In the low-salt group, PWV remained unchanged (6.6 ± 1.0 m/s to 6.5 ± 1.1 m/s, *P* = 0.27) following amiloride. Twenty-four–hour systolic BP decreased significantly from 135 ± 11 to 130 ± 13 mm Hg (*P* = 0.03), whereas the decrease in diastolic BP was not statistically significant (85 ± 9 vs. 83 ± 10 mm Hg; *P* = 0.10). We observed a modest positive association between PWV and 24-hour systolic BP during the study (Spearman ρ = 0.43, *P* < 0.01, Supplementary Figure S1). Individual changes in PWV and 24-hour BP after salt intake and amiloride addition are shown in Supplementary Figures S2–S4.

**Table 3:** Outcomes before and after initiation of amiloride in the salt group

Outcomes	Salt group *n* = 17	Salt group with amiloride *n* = 15	*P*-value
BP and PWV values			
24-h systolic BP, mm Hg	138 ± 15	135 ± 13	0.30
24-h diastolic BP, mm Hg	87 ± 9	87 ± 5	0.32
Pulse wave velocity, m/s	6.8 ± 1.1	6.6 ± 1.1	0.10
Plasma measurements			
Creatinine, μmol/l	81 ± 21	77 ± 12	0.54
eGFR, ml/min per 1.73 m^2^	87 ± 22	88 ± 21	0.20
Urea, mmol/l	4.9 ± 1.3	5.4 ± 1.2	0.05
Sodium, mmol/l	141 ± 1	140 ± 2	0.07
Potassium, mmol/l	4.0 ± 0.2	4.3 ± 0.2	0.01
Bicarbonate, mmol/l	25.9 ± 2.1	24.8 ± 1.9	0.36
Chloride, mmol/l	105 ± 2	105 ± 3	0.37
Renin, pg/ml^a^	11.3 (8.1–14.8)	21.0 (16.3–28.4)	< 0.01
Aldosterone, pg/ml^a^	167 (112–193)	414 (329–474)	< 0.01
24-h urine measurements			
Volume, ml	2428 ± 999	2454 ± 1166	0.77
pH	6.2 ± 0.4	6.5 ± 0.6	0.02
Osmolarity, mOsm/kg	363 ± 186	392 ± 232	0.56
Creatinine, mmol/d	13 ± 5	11 ± 4	0.54
Urea, mmol/d	342 ± 121	372 ± 190	0.67
Sodium, mmol/d	135 ± 55	135 ± 40	0.99
Potassium, mmol/d	76 ± 23	69 ± 31	0.40
Chloride, mmol/d	135 ± 46	126 ± 36	0.51
Albumin, mg/d^a^	20 (10–38)	20 (10–40)	0.36

eGFR, estimated glomerular filtration rate; PWV, pulse wave velocity.

a Median (Q1–Q3).

**Table 4:** Outcomes before and after initiation of amiloride in the low salt group

Outcomes	Low salt group *n* = 17	Low salt group with amiloride *n* = 17	*P*-value
BP and PWV values			
24-h systolic BP, mm Hg	135 ± 11	130 ± 13	0.03
24-h diastolic BP, mm Hg	85 ± 9	83 ± 10	0.10
Pulse wave velocity, m/s	6.6 ± 1.0	6.5 ± 1.1	0.27
Plasma measurements			
Creatinine, μmol/l	80 ± 15	81 ± 15	0.09
eGFR, ml/min per 1.73 m^2^	89 ± 19	88 ± 19	0.09
Urea, mmol/l	5.1 ± 1.8	5.7 ± 1.9	0.01
Sodium, mmol/l	140 ± 3	138 ± 3	< 0.01
Potassium, mmol/l	4.1 ± 0.3	4.4 ± 0.2	< 0.01
Bicarbonate, mmol/l	27.6 ± 1.8	26.1 ± 2.3	0.01
Chloride, mmol/l	102 ± 3	102 ± 2	0.63
Renin, pg/ml^a^	17.0 (16.0–26.2)	44.6 (32.1–48.3)	< 0.01
Aldosterone, pg/ml^a^	230 (136–305)	577 (526–1088)	< 0.01
24-h urine measurements			
Volume, ml	2519 ± 662	2625 ± 799	0.42
pH	6.3 ± 0.5	6.2 ± 0.5	0.57
Osmolarity, mOsm/kg	219 ± 42	270 ± 93	0.16
Creatinine, mmol/d	11 ± 4	11 ± 3	0.78
Urea, mmol/d	298 ± 125	335 ± 124	0.19
Sodium, mmol/d	52 ± 32	57 ± 30	0.56
Potassium, mmol/d	65 ± 27	71 ± 24	0.27
Chloride, mmol/d	54 ± 22	60 ± 26	0.47
Albumin, mg/d^a^	15 (10–30)	20 (10–35)	0.26

eGFR, estimated glomerular filtration rate; PWV, pulse wave velocity.

a Median (Q1–Q3).

### Effect on Blood and Urine Parameters

Changes in plasma and urine parameters during the placebo or salt phase and the amiloride phase are shown in Table 2, Table 3, Table 4. During the placebo or salt phase, salt supplementation increased plasma sodium (141 ± 1 vs. 140 ± 3 mmol/l; *P* = 0.06), plasma chloride (105 ± 2 vs. 102 ± 3 mmol/l; *P* < 0.01), and reduced plasma bicarbonate (25.9 ± 2.1 vs. 27.6 ± 1.8 mmol/L; *P* = 0.02) compared with the low-salt group (Table 2). Plasma renin and aldosterone levels were significantly lower in the group that received salt. During the amiloride phase, several biochemical changes occurred in both groups (Tables 3 and 4). In the salt group, amiloride increased plasma potassium (4.0 ± 0.2 vs. 4.3 ± 0.2 mmol/l; *P* = 0.01), plasma urea (4.9 ± 1.3 vs. 5.4 ± 1.2 mmol/l; *P* = 0.05), and urine pH (vs. 6.2 ± 0.4 vs. 6.5 ± 0.6; *P*=0.02), with a nonsignificant reduction in plasma sodium (141 ± 1 vs. 140 ± 2 mmol/l; *P* = 0.07). In the low-salt group, amiloride also increased plasma potassium (4.1 ± 0.3 mmol/l 4.4 ± 0.2 ; *P* < 0.01) and plasma urea (5.1 ± 1.8 vs. 5.7 ± 1.9 mmol/l; *P* = 0.01), while reducing plasma sodium (140 ± 3 mmol/l vs. 138 ± 3; *P* < 0.01) and bicarbonate (27.6 ± 1.8 mmol vs. 26.1 ± 2.3/L; *P* = 0.01). In both groups, amiloride increased plasma renin and aldosterone levels. Circulating markers of endothelial function and inflammation, including endothelin-1, intercellular adhesion molecule-1, vascular cell adhesion molecule-1, and vascular endothelial growth factor, remained unchanged across all study phases in both groups (Supplementary Table S3).

### Safety and Adverse Events

Two adverse events occurred during the study. One participant in the salt group developed abdominal pain because of a possible kidney cyst bleed. One participant in the low-salt group developed asymptomatic hyponatremia (plasma sodium 129 mmol/l) during the run-in phase, which resolved after fluid restriction. No serious adverse events or suspected unexpected serious adverse reactions occurred. No episode of hyperkalemia occurred in either group.

## Discussion

In this randomized controlled trial, increasing sodium intake from a low-salt diet to levels comparable to average Western consumption resulted in a significant increase in vascular stiffness and 24-hour systolic BP in adults with early ADPKD. Short-term treatment with amiloride did not reverse these salt-induced changes. In contrast, amiloride significantly lowered systolic BP under low-salt conditions. Together, these findings show that vascular function and BP in ADPKD is sensitive to dietary sodium intake, even within ranges commonly encountered in the general population.

The PWV values observed in our cohort are consistent with previous reports of early vascular dysfunction in ADPKD, in which adults with preserved kidney function show PWV values more typical of older or higher-risk populations.^15^^,^^16^ The clinical significance of the observed salt-induced PWV change is difficult to quantify. Nevertheless, a meta-analysis indicates that each 1 m/s higher aortic PWV is associated with a 15% greater risk of cardiovascular events and mortality,^17^^,^^18^ though this association was derived from between-person comparisons over years of follow-up and cannot be directly extrapolated to the small, short-term within-person change observed here; whether such transient shifts have any bearing on long-term cardiovascular risk is unknown. An interesting observation is that PWV and 24-hour systolic BP showed only a modest positive association during the study (Spearman ρ = 0.43, *P* <0.01). The concomitant increase in BP following salt intake may therefore have contributed, at least in part, to the observed increase in PWV; individual changes in PWV and BP did not consistently correspond. As the Mobil-O-Graph derives PWV using a transfer function incorporating brachial pressure waveform characteristics, a degree of measurement dependency between the 2 cannot be fully excluded. This suggests that arterial stiffness and BP were related but not interchangeable outcomes, though firm conclusions cannot be drawn from this finding alone.

The BP-lowering effect of amiloride was observed only under low-salt conditions, suggesting that dietary sodium restriction potentiates the antihypertensive response to ENaC inhibition. This is consistent with previous evidence that sodium restriction enhances BP control during antihypertensive treatment.^19^ Additionally, the preceding period of sodium restriction may also explain the relatively low BP observed at baseline, despite withdrawal of antihypertensive medication. The effect of amiloride in this study was modest and did not reach statistical significance over the short treatment period. In a 24-week randomized trial in early ADPKD, spironolactone reduced BP without clearly improving arterial stiffness, but sodium intake was not controlled in this study.^20^ Our study extends the literature by showing that higher sodium intake alone increases arterial stiffness. Whether longer-term amiloride treatment can further decrease arterial stiffness in ADPKD remains to be determined.

ENaC is expressed both in the kidney and the vasculature.^21^ Our study does not allow separation of the vascular ENaC effects from its renal effects. Amiloride induces natriuresis and modest volume contraction, as reflected by the observed rises in plasma urea, renin, and aldosterone, as well as BP changes and increases in plasma potassium; therefore, any vascular signal may reflect a combination of direct endothelial effects and indirect hemodynamic and hormonal effects. The compensatory rise in renin and aldosterone observed following amiloride may have counteracted its direct endothelial benefits, given the known vascular effects of aldosterone acting through mineralocorticoid receptors in vascular smooth muscle cells and fibroblasts.^22^ Future studies incorporating an active natriuretic comparator (e.g., thiazide diuretic) would be needed to determine whether ENaC inhibition provides vascular benefit beyond kidney sodium losses.

This study has several strengths and limitations that merit consideration. Strengths include the randomized, double-blind study design, and the use of 24-hour ambulatory BP monitoring in a well-characterized ADPKD cohort with preserved kidney function. Limitations include the short intervention period, which precludes assessment of long-term vascular remodeling. PWV was assessed using a brachial cuff–based oscillometric device, and values are not directly interchangeable with carotid–femoral PWV; however, this method has been validated and applied in multiple cardiovascular and kidney disease studies.^23^, ^24^, ^25^ Only 15 participants in the salt group completed the amiloride phase, potentially limiting the statistical power to detect a more subtle effect on PWV. However, whether such a small additional reduction in PWV would be clinically meaningful is uncertain. In addition, the amiloride phase was open-label and lacked an active diuretic comparator, limiting conclusions regarding the specificity of ENaC inhibition on vascular outcomes.

In conclusion, vascular stiffness and BP in early ADPKD are sensitive to dietary sodium intake. Although amiloride did not reverse salt-induced arterial stiffness, it significantly lowered BP under low-salt conditions, suggesting that sodium restriction potentiates the antihypertensive effect of ENaC inhibition and should be considered an integral component of BP management in early ADPKD.

## Disclosure

All the authors declared no competing interests.

## Supplementary Material

Supplementary file (PDF)

**Figure S1**. Association between pulse wave velocity and 24-hour systolic BP.

**Figure S2**. Individual changes in pulse wave velocity and 24-hour sodium excretion.

**Figure S3**. Individual changes in 24-hour systolic BP and 24-hour sodium excretion.

**Figure S4**. Individual changes in 24-hour diastolic BP and 24-hour sodium excretion.

**Table S1**. Changes from baseline after initiation of salt or placebo capsules.

**Table S2**. Changes after initiation of amiloride.

**Table S3**. Changes in (vascular) biomarkers at different timepoints.

**CONSORT Checklist.**
